# Palliative and Critical Care: Their Convergence in the Pediatric Intensive Care Unit

**DOI:** 10.3389/fped.2022.907268

**Published:** 2022-06-10

**Authors:** Siti Nur Hanim Buang, Sin Wee Loh, Yee Hui Mok, Jan Hau Lee, Yoke Hwee Chan

**Affiliations:** ^1^Pediatric Palliative Care Service, Department of Pediatric Subspecialities, KK Women's and Children's Hospital, Singapore, Singapore; ^2^Children's Intensive Care Unit, Department of Pediatric Subspecialties, KK Women's and Children's Hospital, Singapore, Singapore

**Keywords:** pediatric intensive care unit (PICU), integrative models, critical care, palliative care, framework

## Abstract

Palliative care (PC) is an integral component of optimal critical care (CC) practice for pediatric patients facing life-threatening illness. PC acts as an additional resource for patients and families as they navigate through critical illness. Although PC encompasses end of life care, it is most effective when integrated early alongside disease-directed and curative therapies. PC primarily focuses on improving quality of life for patients and families by anticipating, preventing and treating suffering throughout the continuum of illness. This includes addressing symptom distress and facilitating communication. Effective communication is vital to elicit value-based goals of care, and to guide parents through patient-focused and potentially difficult decision-making process which includes advanced care planning. A multidisciplinary approach is most favorable when providing support to both patient and family, whether it is from the psychosocial, practical, emotional, spiritual or cultural aspects. PC also ensures coordination and continuity of care across different care settings. Support for family carries on after death with grief and bereavement support. This narrative review aims to appraise the current evidence of integration of PC into pediatric CC and its impact on patient- and family-centered outcomes. We will also summarize the impact of integration of good PC into pediatric CC, including effective communication with families, advanced care planning, withholding or withdrawal of life sustaining measures and bereavement support. Finally, we will provide a framework on how best to integrate PC in PICU. These findings will provide insights on how PC can improve the quality of care of a critically ill child.

## Introduction

Palliative care (PC) is recognized to be an integral component of optimal critical care (CC) practice for children facing life-threatening illness (LTI) or life limiting illness (LLI). Although it encompasses end of life (EOL) care, PC is most effective when integrated early alongside disease-directed and curative therapies. Professional organizations such as World Health Organization and the American Academy of Pediatrics endorse early integration of PC in management of seriously ill children, regardless of whether the patient is receiving disease-directed therapy and their expected outcome (1, 2). The primary goal of PC is to enhance quality of life, reduce suffering, optimize function and support both patient and families.

Pediatric intensive care units (PICUs) care for children with serious illnesses, complex medical conditions and technology dependence. While overall PICU mortality is low and declining, up to 80% of all inpatient pediatric deaths occurs in the PICU setting, often preceded by withdrawal of life-sustaining therapy (3–5). Improved PICU survival rate has also resulted in more acquired morbidities and chronic complex conditions (CCC) in survivors, shifting the focus of CC from aggressive life-sustaining therapy to one that maintains comfort and preserves quality of life in this group of patients (6). Timely and optimal management of distressing symptoms is important to reduce patient's suffering. It is also imperative to address families' emotional, psychological and spiritual distress while they make difficult decisions for their critically ill child. For these reasons, early integration of PC with CC is recommended (7). Despite its established benefits, PC utilization for critically ill children remains low with considerable variability across institutions (8, 9). This highlights the need to standardize integration and utilization of PC into CC.

This narrative review aims to summarize current literature to describe different models for integrating PC into CC and its impact on patient and family-centered outcomes. We will highlight the skills in PC that is required in PICU and the range of needs which arise from these children and their families. Finally, we will provide a framework on how best to integrate PC in the CC setting.

## Challenges of Pediatric Palliative Care

Pediatric palliative care (PPC) is a multidisciplinary clinical approach which delivers patient and family centered care to children with LLI or LTI to minimize suffering while maximizing quality of life (1, 10). Although PPC is a rapidly growing field, PC for adults is comparatively far more established. There are several fundamental differences between the pediatric and adult population which preclude the generalisability of adult PC on children. Table 1 summarizes the differences between adult and palliative population and its impact on PPC (6, 11–14).

**Table 1:** Differences between adult and pediatric palliative patients.

**Domain**	**Adult population**	**Pediatric population**	**Implication to PPC**
**Patients characteristic**	Less diverse population • Age range tends to be narrower.	More diverse population • Age range tends to be wider (spanning from *in-utero* though young adulthood).	Management and communication need to be constantly tailored to child's level of comprehension, emerging autonomy, parental views and child's condition.
**Underlying diagnosis and comorbidities**	• Malignancy is the most common diagnosis.	• Large variety of congenital and acquired conditions with unknown trajectories and evolving treatment goals. • Growing proportion of children having complex chronic conditions	PPC specialists need a broad understanding of pediatric conditions and be able to address both chronic and acute end of life symptoms It is common for PPC specialists to provide symptom control and decision-making support while potential treatment is still being pursued.
**Duration of PC needed**	• Average duration of survival after initiation of PC: 1–3 months.	• Survivorship after initiation of PC can range from hours to years.	PPC should begin at time of LTI diagnosis and continue throughout disease trajectory.

*PPC, pediatric palliative care; LTI, life threatening illnesses*.

The death of a child has been described as the most stressful of life events with significant implications (15). Parental grief after the loss of a child is more intense and prolonged compared to grief experienced by adults who has suffered the loss of a spouse or parent (16). Many studies reported that many bereaved parents suffer from long-standing mental health issues such as complicated grief, depression and post-traumatic stress disorder (PTSD) (17–20). Bereaved parents are also shown to have increased health risk for cancers, type 2 diabetes, myocardial infarction and acute illnesses (21–24). For these reasons, early PPC is advocated to provide additional support for both patient and families.

A growing body of literature describe direct benefits of PPC for patients, families and staff. Despite this, the adoption of PPC is still suboptimal. A prospective international multicenter study on PICU mortality showed that patients who died in PICU were less likely to have a DNR or PC consult compared to patients who died in another inpatient setting (25). Systemic integration of PPC into CC practice will likely improve this situation. This is demonstrated in a retrospective study in Taiwan where a standardized approach to EOL care resulted in increased willingness to accept withdrawal of life-sustaining interventions and lowered PICU care intensity, such as PICU utilization and use of catecholamines infusion in patients with the DNR status (26).

### Impact of Palliative Care Interventions

#### Pain and Symptom Management

Pain, from the disease or interventions, is the most common symptom experienced by critically ill children in PICU (27, 28). Other commonly experienced symptoms are nausea, dyspnea and delirium. There are numerous barriers that may contribute to under-reporting of these symptoms. This includes communication difficulties by PICU patients due to severity of illness, unrecognized delirium, neurocognitive impairment and presence of invasive support such as endotracheal tube. Pain assessment in the pediatric population is also more challenging than in adults because patients of different age groups express pain differently. A wide range of pain rating scales for different age groups and verbal skills are readily available to achieve consistency of pain assessment (29). Initiatives to improve EOL care in PICU should include raising awareness of pain as a vital sign and standardizing guidelines for symptoms management.

The management of distressing EOL symptoms is of upmost importance in PC. Retained memories of unrelieved EOL symptoms have negative impacts on bereaved parents and siblings (30). A high index of suspicion and close monitoring assist with identification of symptoms, which can be quickly followed by aggressive interventions in collaboration with subspecialties like acute pain team and PC team. Early engagement of PC team positively impact patient and family-centered outcomes by facilitating better pain and EOL symptoms management. Alleviation of physical EOL symptoms also enables PC team to form a good rapport and better negotiate domains of psychological and spiritual care with the family and patient.

#### Effective Communication

Effective communication is an essential pillar of good pediatric CC. Parents of PICU patients are often overwhelmed with medical concepts and uncertainties and are required to make high-stake decisions for their child. Many PICU physicians and subspecialists function on a roster basis, making continuity of care challenging (7). In a qualitative study, many parents reported that the sheer number of physicians and the coordination of communication added on to their emotional burden and eroded their confidence as they needed to seek clarifications (31). PC specialists can act as a constant and strengthen the team's ability for effective communication in such instances.

The high stress environment of the PICU may predispose to conflicts between physician-family, among physicians and within family (32). Conflicts compromise quality of care and contributes to physicians' burnout (33). Commonly cited reasons for physician-family conflict are disagreement over care plans and poor communication (32, 34). Sources of conflict among physicians include disagreement in medical decisions such as pain management, lack of leadership and undervaluing each other's role in a multidisciplinary team, all of which fall under the umbrella of poor communication (35). Palliative specialists can help neutralize tension between all parties and redirect the focus toward advocating for the child's best interest.

End of life discussion in PICU is a delicate and challenging process for physicians, with uncertainty around prognosis of many pediatric conditions adding to the complexity of it. Even though communication is one of the core skills of PICU physicians, many are uncomfortable with EOL discussion and may delay these important conversations (36). This delay can result in missed opportunities for identification of emotional issues and negatively impair healing for the family (37). A cross-sectional study of family conferences held in the PICU of Children's National Hospital, United States reported that nearly three quarters of family conferences and 79% of physician speech was medically focused (38). This study also reported that a higher patient-centeredness score was associated with higher patient satisfaction (38). The family-centered model of PC helps forge a beneficial and supportive partnership between families and physicians.

#### Advanced Care Planning

Advanced Care Planning (ACP) aims to facilitate early planning of treatment goals, including EOL care, through professionally facilitated discussions with patients and families (39). Positive impacts of pediatric ACP include higher rating of EOL care in patients, decrease negative emotions in parents and enabling parents to be better informed and certain about their decisions (40, 41). A study on bereaved parents reported that all parents felt that ACP was important even though only 61% of the parents had finalized ACP prior to their child's death (42).

The answer to when, how and who to initiate ACP remains controversial. A study on clinical providers' attitudes on ACP identified unrealistic parent expectations, differences between clinical and patient/parent understanding of prognosis and lack of parent readiness as the top 3 barriers to ACP discussion (43). Despite having clarity of the barriers, 71% of the respondents believed that ACP happened too late in the patient's clinical course (43). Naturally, many physicians feel insecure about discussing ACP as they are worried about burdening families and destroying the therapeutic alliance with parents (44). PC specialists can step in to share this burden and ensure timely discussion of ACP in a sensitive manner. It is, however, important that ACP discussion should not be owned by a particular physician and should be shared by the entire medical care team.

#### Withdrawal, Withholding of Life Sustaining Therapy or Non-escalation

A framework by the Royal College of Pediatrics and Child Health, United Kingdom states that there are three sets of circumstances when withholding, withdrawal or non-escalation of life sustaining interventions (WWNLST) can be considered (i) when life is limited in quantity, (ii) when life is limited in quality, (iii) lack of ability to benefit (45). Transition in goals of care from curative to comfort should be made by clinical teams in partnership, and with the agreement of, the parents and patient. PPC can work together with PICU physicians to identify patients suitable for WWNLST and aid in timely open discussion with the family to achieve consensus. A large single center retrospective study in Spain reported that WWNLST was more frequently facilitated in suitable patients after the development of a PC unit (46).

The practice of WWNLST remains highly variable despite many published recommendations (47, 48). Poorly handled WWNLST can lead to confusion and distress for the patient, family and medical staff. The role of PC is to formulate a carefully thought-out plan, from planning to post withdrawal of care, to ensure a smooth process and an optimal experience for all involved stakeholders.

Compassionate extubation at home (CEAH) is a valuable service that PICU can offer and facilitate. The familiarity and comfort of home help families achieve a higher level of satisfaction and comfort with their child's EOL care (49). Medical staff involved in CEAH also reported it to be valuable despite its complex orchestration (50). Despite being resource intensive and logistically challenging, reports have reaffirmed the feasibility of CEAH in the pediatric population with positive outcomes (51, 52). A recently published framework detailing processes from preparation to follow through acts as a good reference for PICU intensivists in their provision of CEAH as an option of EOL care (53).

#### Bereavement Care Services

The death of a child can lead to long-term adverse effects on parental and siblings' physical and psychological health (54, 55). Data also suggest that bereaved parents have higher mortality rates (56). The goals of bereavement support are to facilitate healing and adjustment of bereaved parents after the death or their child so that they can continue to live normal and meaningful lives, and also to carry out early intervention for individuals at risk of negative bereavement reactions (57). Despite the established benefits, hospitals lack coordinated and standardized bereavement programs (58).

A systematic review identified five key components of pediatric bereavement: (i) acknowledgment of parenthood and child's life; (ii) establishing keepsakes, (iii) follow-up contact, (iv) education and information, and (v) remembrance activities (59). However, only four out of 12 studies reported interventions that commenced before the death of the child, inconsistent with bereavement theories of facilitating the transition of parents toward a new reality (59). A qualitative study conducted in the United States reported that five out of nine bereaved parents experienced feelings of abandonment by the medical team after the death of their child, with some parents verbalizing their wish for follow up meeting or support (57). These highlight the need for improvement and standardization of bereavement care.

An anecdotal report by a PPC physician on primary medical providers after redirection of patient's care plan toward comfort stated “sudden loss of power of prescription” and “assumption that bereavement should be delegated to other team members” as challenges primary providers faced (60). Hence, PC specialists can help to ensure that important components of pediatric bereavements are met and to empower primary physicians in providing bereavement care, reducing the risks of adverse effects associated with the death of a child.

#### Other Outcomes

A systematic review of adult controlled trials reported a reduction in relative risk of ICU admission and ICU length of stay by 37 and 26%, respectively, in patients who received PC interventions and ACP discussion (61). A pediatric retrospective study conducted at St.Jude Hospital, United States reported that children who received PC intervention were significantly less likely to die in PICU and to receive invasive treatment (62). These outcomes have important economic implications and reduce the financial burden of some families. Indeed, an adult cohort study demonstrated that patients who received PC incurred significantly lower costs as a result of reduced length of hospital stay and number of investigations performed compared to patients who received usual care (63).

### Integrating Palliative Care Into PICU: Models of Care

**Figure 1:**
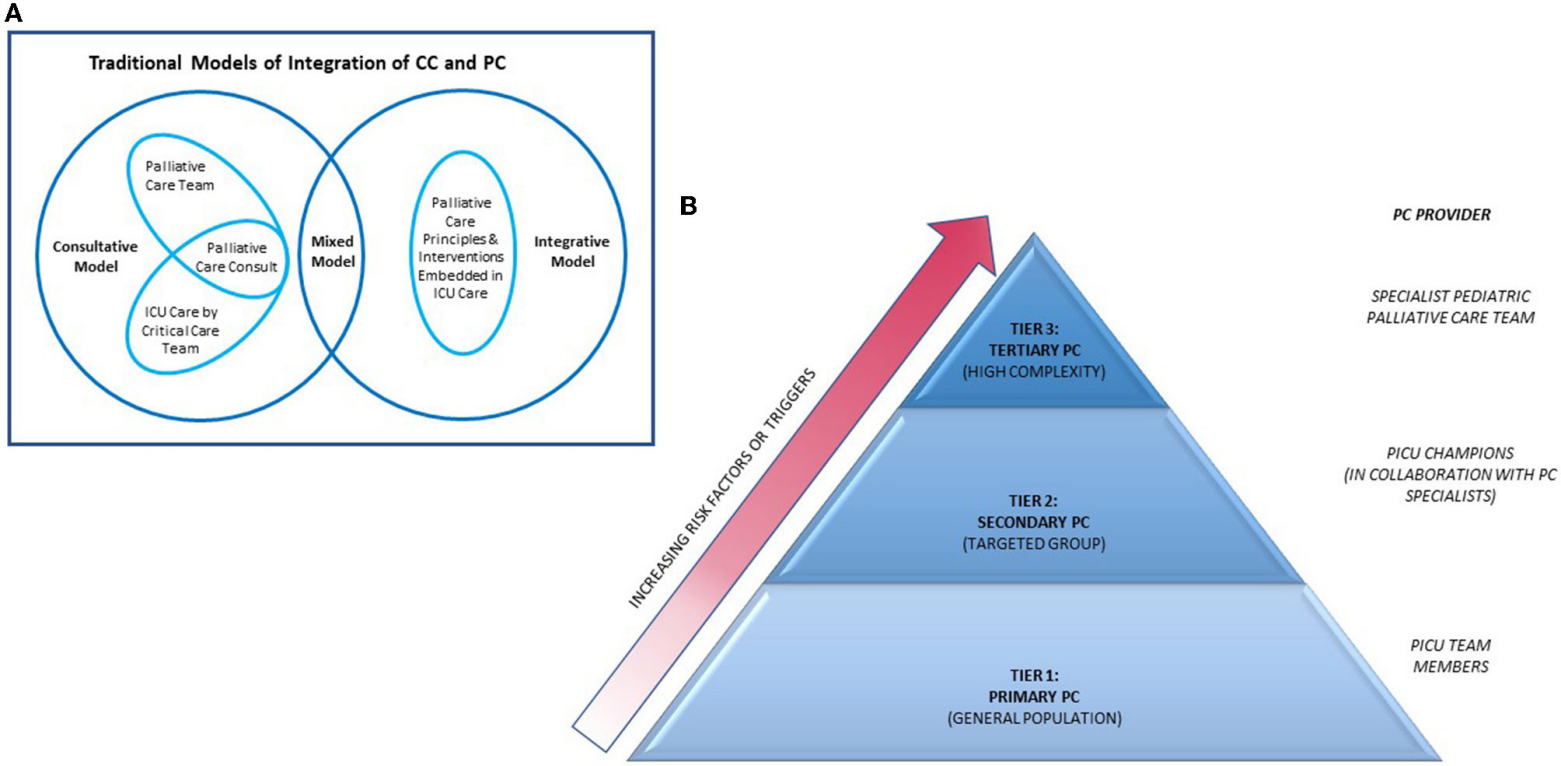
Models of integration of PC into ICU. PC, palliative care; CC, critical care; ICU, intensive care unit. **(A)** Diagram depicting interactions of traditional models of integration of PC into ICU. Adapted from Nelson et al. (64). **(B)** Pyramid model of integrating of PC into ICU. Source—Public domain and adapted from Rothschild et al. (65).

The integration of PPC in PICU is largely extrapolated from adult models. The traditional models for PC-CC integration can be broadly classified into “integrative,” “consultative,” and “mixed” models (64). The integrative model embeds standardized PC principles and interventions into daily CC practice by the ICU team for all patients and families facing critical illness. The consultative model incorporates the involvement of specialist PC team on a needs basis, reserved for those at highest risk for poor outcomes. Successful implementation of this model includes the use of clinical triggers for expert PC consult. Mixed models would feature aspects of both integrative and consultative models (Figure 1).

More recently, a tiered approach for PC-CC integration has been described (Figure 1) (65). In this model, interventions are categorized into primary, secondary and tertiary PC, with increasing PC specialists involvement across the levels. Primary PC is the provision of evidence-based PC interventions by critical care physicians. This is useful in institutions where dedicated PC teams are not available. Examples of primary PC interventions can be found in the Initiative for Pediatric Palliative Care (IPPC) curriculum which highlights six core constituents of quality PPC: holistic care of the child, support of family unit, involvement of the family and child in decision-making, communication and planning of care, treatment of pain and other symptoms, continuity of care and support of grief and bereavement (66). PICU physicians are also equipped with knowledge about EOL issues including pronouncing death and discussing need for autopsies. However, delivery of primary PC can be highly variable and is dependent on resources, manpower and critical care physicians' knowledge on PC. An international multicentre cross-sectional study including 34 PICUs of varying socio-economic settings reported heterogenous and incomplete fulfillment of IPPC domains in their delivery of primary PC, with better adherence in higher income groups and units with shorter shift lengths (67).

Secondary PC uses ICU-based champions who receive additional PC training through courses and subspecialty rotations. These ICU champions strengthen the delivery of PC in ICU by spearheading PC-based training for other ICU staff, advocating for earlier PC subspecialty involvement in suitable patients and also improving PC *via* quality improvement initiatives and protocols development. A recent report describes the integration of a pediatric palliative care-champion (PPCC) based model into the cardiac ICU in Boston Children's Hospital, United States (68). The PPCC model is expected to be more sustainable than other PC-CC integration models as the workload is shared with overextended subspecialty PC services, hence relieving the strain on PC teams while allowing early integration of PC principles in the ICU. However, provision of secondary PC will require commitment from ICU providers and a robust PPC team to support program development and education.

Tertiary PC involves consultation of a subspecialty PC team as an additional resource. This is helpful in specific situations where PC team can facilitate more difficult communications, support complex decision making in the face of uncertainty or conflict while providing both emotional and spiritual support for both patient and family and assist with managing difficult symptoms. Added benefits include ensuring continuity in goals of care and care coordination across multiple providers and settings.

Defining clinical triggers for PC consultations ensures that palliative consults are made appropriately. In PICUs, common triggers criteria include baseline patient characteristics (e.g., extreme prematurity), selected acute or life-limiting diagnoses (e.g., severe traumatic brain injury, Trisomy 13), resource utilization based criteria (e.g., ECMO duration, number of ICU admissions over time), social risk factors or failure of initial ICU efforts to address PC needs of patients and families (64). However, variability in resources and systems of care limits the use of a fixed set of trigger criteria across institutions. Adaptation of triggers mapped to institution resources and needs is a more logical approach (69).

Choosing to adopt any of the above models can be an important initial step toward an initiative to incorporate PC practice into the PICU. Both primary and secondary PC have the same characteristics as the integrative model while tertiary PC is most alike to the consultative model. In reality, there is usually a large degree of overlap between models and no one model can suit the demands of all institutions. Careful and realistic assessment of available resources, attitude of stakeholders, cultural and value system of the institution would be required to find the best fit.

## Conclusion

The integration of PC to CC has many positive impacts on patient and family-centered outcomes and is becoming the standard for high-quality care of critically ill children. PC also ensures both coordination and continuity of care across different care settings are met. Several models of integration have been proposed but the model of choice should be tailored to available resources, attitude of stakeholders, cultural context and value system of the institution.

## Author Contributions

SB and SL wrote sections of the manuscript. YM, JL, and YC contributed additional resources/journals and advice regarding content. All authors contributed to manuscript revision, read, and approved the submitted version.

## Conflict of Interest

The authors declare that the research was conducted in the absence of any commercial or financial relationships that could be construed as a potential conflict of interest.

## Publisher's Note

All claims expressed in this article are solely those of the authors and do not necessarily represent those of their affiliated organizations, or those of the publisher, the editors and the reviewers. Any product that may be evaluated in this article, or claim that may be made by its manufacturer, is not guaranteed or endorsed by the publisher.
